# Stressors in Indoor and Field Brazilian Soccer: Are They Perceived as a Distress or Eustress?

**DOI:** 10.3389/fpsyg.2021.623719

**Published:** 2021-05-20

**Authors:** Maria Regina Ferreira Brandão, Luis Felipe Polito, Vania Hernandes, Mariana Correa, Ana Paula Mastrocola, Daniel Oliveira, Alessandra Oliveira, Larissa Moura, Marcelo Villas Boas Junior, Daniela Angelo

**Affiliations:** Master’s and Doctoral Programme in Physical Education, São Judas Tadeu University, São Paulo, Brazil

**Keywords:** eustress and distress, perception, performance, soccer (football), stress

## Abstract

Soccer players inescapably live under stress during the sportive career, and many real-life aspects of soccer situations operate in the ongoing performance. This study’s main objective was to elaborate the List of Stressors in Professional Indoor and Field Soccer, a self-report instrument designed to measure the impact of 77 soccer situations upon the sport performance. Participants were 138 indoor and field soccer players from the Brazilian Premier League. Each situation was evaluated on a 7-point scale, ranging from the most negative (−3) to the most positive (+3). Data were analyzed according to the players’ perception of the items: distress or eustress and its intensity, and after that, situations perceived as plus −1 and +1 were compared by time in which they were experienced and distributed among five categories established by the literature: Expectations about the Performance, Personal Factors, Competition Aspects, Training Demands, and Relationship with Significant People. Narratives of athletes’ experiences were also used to discuss the results. An Exploratory Structural Equation Modeling using Bi-factorial (BI-ESEM) was employed to assess the factor structure. For the total participants, 49 situations were perceived as distress and 28 as eustress. Using the criteria established *a priori*, the distribution was among the five categories in the remaining 32 situations. Differences in perception between less and more experienced players were found in 11 situations. The results revealed that Brazilian professional soccer players experience various stressful situations. These events are important representations of environmental demands and could predict the performance as they are perceived as eustress or distress. Some of these stressful situations are inherent in sport and others adjacent to the sports system or environment. Coach pressure to win and conflicts with teammates are examples of stressors in-sport, family problems and disputes with press or fans are examples of stressors external to the team, also called peripheral opponents, and showed the relative social influence of significant others in soccer performance. We can conclude that the knowledge of the direction of a given stress situation has important practical implications in preparing athletes and helping them face the performance stressors that are part of soccer daily life.

## Introduction

The term *stress* started to be used only at the beginning of the 20th century. Until then, the term was used in physics to describe the force that tends to deform an object. In 1910, Sir William Osler, a British cardiologist, first suggested that stress contributed to coronary heart disease (Robinson, 2018). Since then, stress has moved from a term in physics to a cultural construct, and numerous studies have been carried out to detect the effects of stress on physical and mental health.

Selye (1976), considered the patriarch of research on stress in human beings, refers to a “General Adaptation Syndrome,” a syndrome due to the presence of generalized individual manifestations of the organism in the face of a harmful stimulus, adaptation by stimulating the body’s defenses, and general because it is produced only by agents that have a widespread effect on large parts of the body.

From the point of view of psychological stress research, the main emphasis of the studies is not on the physiological genesis, but on the psychological genesis of stress, changes in the well-being of individuals, cognitive processes, and appraisal of stressors and their psychological control (Nitsch, 1981; Jones, 1990). According to this conception, the following principles are important: the starting point of the study of stress is in the reciprocal relationship between the individual and the environment, in subjective perception and in the attribution of value judgments that the individual has about the situation of this environment (Folkman and Lazarus, 1984; Lazarus, 2000).

So, what makes a stressful situation is the nature of this circumstance and the interpretation, the way people subjectively perceive and evaluate the situation. This perception depends on the psychic dispositions (attitudes, beliefs, and values) and previous learning. People do not behave passively in the face of environmental stimuli but rather give them personal importance. In the relationship between individual and environment, an intermediate cognitive process is characterized by subjective evaluation, which is considered a decisive point for the emergence of stress (Nitsch, 1981).

In other words, stress is not necessarily debilitating and can facilitate performance. Then, it is vital to research the “directional perception” of the sources of stress, that is, the nature of the individual interpretation of the sources in terms of whether they are positive or negative about the next performance (Jones et al., 1990, 1993; Swain and Jones, 1993; Jones, 1995a; Seligman and Csikszentmihalyi, 2000; Fletcher and Hanton, 2003).

Hypothetically, athletes can report identical sources of stress, but because of variations in “directional perception,” they can differ considerably in their interpretations of the consequences on performance. Parfitt et al. (1990) still claim that the “directional perception” of stress sources can predict sports performance better than just the simple indication of a source.

Soccer players, indoor or field, are inevitably exposed to a potential number of stressors that operate on athletic performance during their sports career (Poulus et al., 2020). Professional players are under considerable stress, and they need to compete for a place on the starting lineup and, once they win the place, they must be able to keep it. Low performance in just one game can take the player to the bench in the next round. The risk of injury is always present and can mean the absence of practice for more or less long periods. The athlete must deal with the stress produced by the so-called peripheral opponents, spectators, the press and family members, and the expectations of the coach and his teammates (Apitzsch, 1994; Teipel et al., 1994; Noblet and Gifford, 2002; Mellalieu et al., 2009; Olmedilla et al., 2019).

During training and competitions, soccer players are faced with a series of requirements, stressors, which can vary in terms of the content and intensity of their effect on sports performance (Jones et al., 1993; Swain and Jones, 1993; Jones, 1995a; Brandão, 2000; De Rose Junior et al., 2004; Zahariadis et al., 2006; Thelwell et al., 2008, Main and Grove, 2009; Garcia-Maás et al., 2010). But, for Urhausen et al. (1998) and De Rose Junior et al. (2004), it is not only during training and competitions that stressful situations occur. They also appear in events inside and outside of sport and influence an athlete’s mental and physical readiness. Thus, an important aspect pointed out in evaluating sport stressors concerns the sources of stressful situations and the perception of these sources’ direction and intensity. The psychological and physiological demands in soccer associated with training, competitions, and the social organization of sport show that performance is a complex phenomenon affected by factors inherent to the sport modality and environmental factors.

A literature review (Cohn, 1990; Scanlan et al., 1991; Samulski and Chagas, 1992; De Rose Junior and Vasconcellos, 1993; VanYperen, 1994; Brandão, 2000; De Rose Junior et al., 2004) showed that there are 5 major sources of stress inherent to various sports: expectations about performance, personal factors, aspects of competition, physical demands, and relationships with significant people and traumatic experiences. The category expectations about performance is composed of two subcategories: goals and pressure. The goal refers to the athlete’s ambition to achieve a certain sporting result and the pressure, the demands of the media, himself, and others that indicate performance expectations, pressure to meet the established goals, “obligation” to win a certain game and to achieve expected results. Personal factors refer to the demands (costs or psychological benefits) of sports practice. This category includes the athlete’s disposition, his psychological and organic state, and other aspects of professional practice such as the employment contract. The competition aspects refer to the events that commonly occur during the competition. A negative stress response occurs when the athlete has feelings or thoughts of concern about some aspect of the game, the conditions of the field, the crowd, the press assessment, etc. Physical demands refer to the role that factors inherent to the sporting event play in the stress process. If the athlete does not have a personal fitness to deal with the demands of training and competitions, consequently there is a risk of failure or a decrease in performance. Finally, the dimension relationship with significant people refers to the extent and nature of the bonds with people that are significant for athletes and that influence their performance. These people are fully involved in the structure, dynamics, and social environment of the sport practiced. Teipel et al. (1994), in a study with soccer players, showed that previous defeats, the influence of the fans, and the unexpected results of the opposing team were motivators and, therefore, facilitators of sports performance. On the other hand, being physically weak, uncomfortable competitive conditions, failed actions at the start of competitions, conflicts with the coach, problems with the referees, the coach’s continued criticism from the bench, failed actions, and the negative criticism of teammates was harmful to the performance.

If there is a positive or negative impact of these stressors on soccer players’ sports performance, they need to be investigated. So, the purpose of this study was six, including (a) development of a *List of Stressors in Professional Indoor and Field Soccer* to facilitate the identification of situations that can cause stress in professional indoor and field players, (b) confirm if the stressful situations can be perceived as both distress and eustress, (c) identify the situations perceived as distress and eustress, (d) check which items make up the five previously established categories (*Expectations about the Performance, Personal Factors, Competition Aspects, Training Demands, Relationship with Significant People*), (e) examine the differences in the perception of the situations by age, and time as professional, and (f) assess the factor structure of the list.

## Materials and Methods

### Participants

A total of 138 indoor and field male soccer players (17 goalkeepers, 41 defenders, 47 midfielders, and 33 forward) from the Brazilian Premier League participated in the study (age = *24.46* years, *SD* = *3.93*; sports experience = *11.25* years, *SD* = *4.61*, years as a professional player = *5.62* years, *SD* = *3.77*, and years of soccer practice = *11,68*, *SD* = *4,71* years). The participants comprised players from four different clubs, including Palmeiras, Grêmio, Internacional, and Paulista (Jundiaí). As professional players, they train an average of 10 h per week of football *per se* and 8 h of physical preparation. In addition, in the main championship of the country they compete twice a week, on Wednesdays and Sundays. It is essential to highlight that from all the participants, 18 field soccer players had played for the Brazilian National team.

### Measurements

(1) Socio-demographic and background information.

We assessed age, sports experience, years as a professional player.

(2) Measurement of the stress situations in indoor and field soccer.

### List Development

The list of football situations was designed based on the Life Events Checklist, the dominant method used by researchers in the last 50 years (Dohrenwend, 2006), defined as occurrences, fundamentally critical environmental incidents, that were likely to bring about readjustment-requiring changes in people’s usual activities. Moreover, it was elaborated considering the theoretical review of stress in soccer (Frester, 1976; Samulski and Chagas, 1992, 1996; Teipel, 1993; Brandão, 2000). To develop an initial list of soccer situations, a focus group composed of two specialist soccer coaches and five professional players (three field soccer and two indoor soccer) besides the main researcher of this study was created. The focus group’s objective was to help reflect not only on the situations they considered important but also on the different points of view presented and the strength of each situation to be part of the list of stressful situations in soccer. The group was gathered three times to achieve this goal. The focus group listed 72 situations that ranged from the regular activities involved in the football environment, training, competitions, on the one hand, to the relationship with significant people, such as coaches, referees, teammates and the press, on the other.

After that, five event categories defined *a priori* according to the literature review were used to classify the 72 items. Some examples of events for each of the five categories are: for Category 1: *Expectations about the Performance* (Being the favorite, Coach pressure to win); Category 2: *Personal Factors* (Not sleeping well the night before the competition, Being with the contract already due or close to its maturity date); Category 3: *Competition Aspects* (Playing against a hostile audience, Not scoring a goal that was practically attained); Category 4: *Training Demands* (Being blatantly dribbled during tactical training, training in two periods); Category 5: *Relationship with Significant People* (Having problems or conflicts with the coach, Being jeopardized by the referees).

For the structuring of a procedure that would provide the recording of the conditions of the situations by the athlete, it was interesting to know not only which situations were experienced as distress or eustress but, at the same time, how intensely each situation could act as a disturbance or, on the contrary, as a stimulus to the athletic performance. To this end, a seven-level scale was developed, with three dimensions (eustress, distress, and a performance-neutral dimension). Each dimension had three levels of intensity. Thus, 1, 2, 3 identify the positive or negative impact intensity of the situation to the performance, as follows: +1 (a small amount of positive impact); +2 (a moderate amount of positive impact); +3 (a huge amount of positive impact); −3 (a huge amount of negative impact; −2 (a moderate amount of negative impact); −1 (a small amount of negative impact). The number 0 is the center of the scale and means that the situation neither stimulates positively nor negatively the performance.

The events list was tested in an exploratory study with 24 players to verify the clarity of the items’ instructions and phrasing, the item suitability, and the possibility of inclusion, revision, or rejection of each item in the item pool. Seven item events that presented ambiguous responses and one item that referred only to goalkeepers were neglected. Thirteen items were included to increase the instrument’s credibility. The inclusions were based on the testimonies of the players obtained during the application of the exploratory project. This process generated a second version of the list composed of 77 events.

Then, an initial revision of the second version was made by a panel of judges, composed by a Ph.D. in Sport Psychology, specialist in the study of stress in sport and especially in soccer, by the coach of the Brazilian Soccer National Team and his assistant, the goalkeeper coach of one of the evaluated teams, a Ph.D. in sports training and the main researcher of the study that worked with high performance professional soccer teams, including the Brazilian team. Their suggestions were primarily regarding the understanding of the response to items by players with a low level of education. They recommended using graphic symbols (“faces”) that represented the perception of the direction and intensity of stress. Thus, the following symbols appeared in the test body: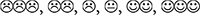 which represent −3, −2, −1, 0, +1, +2, +3, respectively.

Then, these procedures generated the final “*List of Stressors in Professional Indoor and Field Brazilian Soccer*” of this study, which consists of 77 soccer events and five categories, seven items for Category 1; six items for Category 2; 40 items for Category 3 (subdivided into three groups: opponents six items; imminent or real failure eight items; aspects of the game 26 items); 14 items for Category 4; and 10 items for Category 5. It is important to highlight that the item pool was developed so that the language was common to both indoor soccer players and field soccer players. The categories of stressors, definition, and list of the 77 events are shown in Table 1.

**TABLE 1:** Categories of stressors, definition, and list of the 77 events.

Category	Definition	Situations
(1) Expectations about the performance	Refers to the situations related to goals and pressure. The goal refers to the athlete’s ambition to achieve a certain sporting results and the pressure to meet the established goals, “obligation” to win a certain game and achieve expected results.	(5) Being the favorite (6) Establishing high goals (20) Assuming responsibilities inside the team (22) Press pressure (39) Pressure of the other people to win (49) Self-pressure to play well (51) Coach pressure to win
(2) Personal factors	Refers to the demands (costs or psychological benefits) of sports practice. This category includes the athlete’s disposition, his psychological and organic state, and other aspects of professional practice such as the employment contract.	(1) Not being in a good shape (3) Being very nervous (4) Not sleeping well the night before the competition (24) Being with the contract already due or close to its maturity (25) Some other team wanting to book you (73) Lack of psychological preparation
(3) Competition aspects (aspects of the game)	Refers to the events that commonly occur during the competition. A negative stress response occurs when the athlete has feelings or thoughts of concern about some aspect of the game, the conditions of the field, the crowd, etc.****	(2) Staying in the bench and not playing during the game (18) Playing against a hostile audience (19) Playing at home (21) A protracted competition (26) Playing at night (27) Playing in an empty stadium (28) Playing in a rough ground/court (31) Playing in the afternoon (40) Playing a derby (44) Being in the bench and entering during the game (45) Knowing that you are playing minutes before the game starts (52) Knowing in advance that you are going to play (55) Being isolated in a facility on the eve of the match (56) Getting a yellow card (57) Decision by sudden death (58) Playing in the rain (59) Playing injured (60) Playing in the morning (63) Being advised that you are not going to play just before the game (67) Wrong plays in decisive moments (70) Being blatantly dribbled during the game (71) Playing under very warm climate (72) Wrong plays at the end of the game (74) Inadequate technical and tactical preparation (76) Playing an improvised position
(3) Competition aspects (imminent or real failure)	Refers to the situations in which the player has imminent possibilities of failure or which involve a real failure.	(8) Previous defeats (12) Defeats in the beginning of a championship (33) When your team suffers a goal (35) Losing by a dilated score (36) Not scoring a goal that was practically attained (37) Scoring a goal against your own team (38) Losing a penalty (65) Finishing the first half with an adverse score
(3) Competition aspects (opponents’ aspects)	Refers to situations related to opponents in any aspect, behaviors, previous negative experiences, etc.	(7) Playing against unknown opponents (11) Being perplexed with the good performance of the opponents (14) Great superiority of the opponents (17) Having lost previously to the same opponent (30) Being ridiculed by opponent during the game (77) Playing against and aggressive opponent
(4) Training demands	Refers to the role that factors inherent to the sporting event play in the stress process, if the athlete does not have a personal aptitude to deal with the demands of training and competitions, there is a risk of failure or a decrease in performance.	(16) Having bad lodgings and facilities (29) Being blatantly dribbled during tactical training (32) Doing a speed training (34) Stretching (41) Doing a tactical training 1 day before the game (42) Excessive physical training (43) Training early in the morning (47) A long trip (48) Training in 2 periods (53) A hard warm up before the game (62) Doing too much weight exercise (66) A light warm up before the game (68) Pre-season out of the routine premises (69) Too much resistance training
(5) Relationship with significant people	Refers to the extent and nature of bonds with people who are significant to athletes and who influence their performance. These people are fully involved in the structure, dynamics, and social environment of the sport practiced.	(9) Having problems or conflicts with the coach (10) Having problems or conflicts with the teammates (13) Being scolded by a teammate during the game (15) Being jeopardized by the referees (46) Being scolded by the coach during the mid-game interval (50) Press behavior before the game (54) Being scolded by the coach during the pre-game talk (61) Conflicts with the family (64) Receiving threats from the referee during the game (75) Lack of group cohesion

### Data Collection and Procedures

The study was conducted according to international guidelines for ethical principles of scientific research with human beings. The study procedures of this research were also approved by the Human Research Ethics Committee of the University São Judas Tadeu. Indoor and field soccer players from the Brazilian First Division were invited to participate in this study. Upon obtaining participants’ written informed consent, the data were collected, individually, outside training hours, in pre-season periods (preparation period before the competitive period). Participants were encouraged to read each situation and provide honest responses about their effect on the performance and its magnitude. Special emphasis was placed on confidentiality and participation was not mandatory.

After the list evaluation, all athletes undertook a semi-structured interview. The main objective of the individual interviews was to understand the perception of stressful situations from what each player experiences and to give him the possibility to talk about that experience. The interviews also explored how well the items captured the athletes’ experiences about the item’s content. Interviews were audio-recorded and then transcribed verbatim.

### Analysis

All items on the stressor list were analyzed in descriptive statistics and considered if they were perceived as a distress or eustress. Subsequently, a first analysis of the data was made according to the following criteria: the items evaluated between −1 and −3, and +1 and +3 were classified according to five stressors categories defined *a priori*. This interval was chosen because the objective of the study was to identify situations that had a negative or positive impact on performance, and situations assessed close to neutral indicate that according to the perception of the Brazilian players they do not interfere in any way to the performance and, therefore, can be excluded. The one-way analysis of variance (ANOVA) and a *post hoc* test Tukey was then used to compare the players by age (younger, average, and older players) and time as a professional. The level of significance adopted was 0.05.

An Exploratory Structural Equation Modeling using Bi-factorial (BI-ESEM) was employed to assess the factor structure. We chose to use a BI-ESEM model to allow estimates of direct relationships between items and specific and global factors; thus, it is possible to separate the variation attributed to specific factors, from that attributed to the general factor (Holzinger and Swineford, 1937).

Bi-factorial models assume that the covariance between a set of items can be explained by a set of orthogonal factors, including global factor (G-factor; in this case they would be 2 global factors) and specific factors (S-factor). The recent development of bi-factorial rotation for EFA has made it possible to incorporate bi-factorial modeling into the ESEM framework. In BI-ESEM, the G factors were specified separately, outside the rotation process (Arens and Morin, 2017; Howard et al., 2018; Marsh et al., 2020).

To assess the levels of reliability of the latent variables, it was used and the Composite Reliability (CR). Values above 0.7 are considered adequate (Hair et al., 2014).

## Results

**FIGURE 1:**
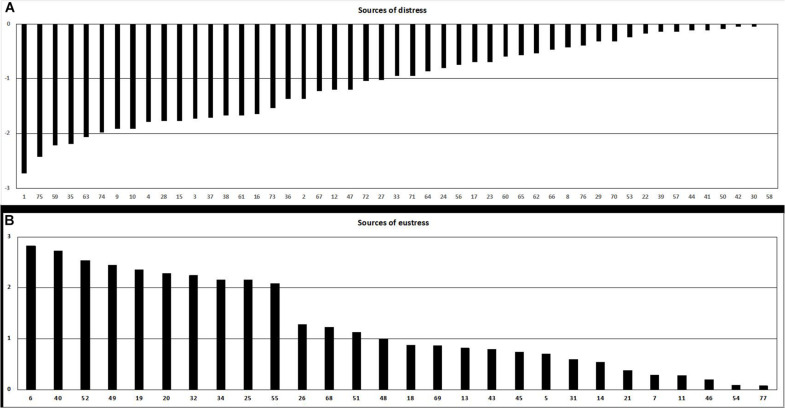
List of stressors perceived as distress **(A)** or eustress **(B)**.

The mean and standard deviation of the 77 items of the list of stressors are shown in Table 2. To better view the results, the events were plotted into a figure (Figure 1) according to the perception of distress (A) or eustress (B). As one can observe, 49 soccer events were perceived as distress and 28 as eustress.

**TABLE 2:** Mean and standard deviation of the 77 items of the list of stressors.

*N* = 138	1	2	3	4	5	6	7	8	9	10	11	12	13	14	15	16
*x*	–2.73	–1.36	–1.73	–1.79	0.69	2.8	0.28	–0.43	–1.91	–1.91	0.27	–1.20	0.81	0.54	–1.77	–1.64
*SD*	0.49	1.45	1.45	1.1	1.55	0.61	1.2	1.59	1.48	1.32	1.33	1.44	1.36	1.78	1.3	1.32

	**17**	**18**	**19**	**20**	**21**	**22**	**23**	**24**	**25**	**26**	**27**	**28**	**29**	**30**	**31**	**32**

*x*	–0.70	0.88	2.30	2.28	0.37	–0.17	–0.70	–0.80	2.15	1.28	–1.03	–1.78	–0.31	–0.05	0.59	2.25
*SD*	1.72	1.63	1.19	1.08	1.39	1.40	1.19	1.49	1.21	1.45	1.38	1.31	0.80	1.32	1.37	1.28

	**33**	**34**	**35**	**36**	**37**	**38**	**39**	**40**	**41**	**42**	**43**	**44**	**45**	**46**	**47**	**48**

*x*	–0.95	2.16	–2.20	–1.37	–1.72	–1.67	–0.14	2.72	–0.12	–0.05	0.68	–0.12	0.74	0.19	–1.20	0.99
*SD*	1.51	1.22	1.24	1.24	1.21	1.18	1.20	0.86	1.46	1.83	1.43	1.71	1.75	1.44	1.13	1.40

	**49**	**50**	**51**	**52**	**53**	**54**	**55**	**56**	**57**	**58**	**59**	**60**	**61**	**62**	**63**	**64**

*x*	2.45	–0.09	1.13	2.53	–0.25	0.08	2.08	–0.75	–0.14	–0.10	–2.22	–0.59	–1.67	–0.54	–2.07	–0.86
*SD*	1.03	0.69	1.55	1.05	1.64	1.36	1.25	1.03	1.21	1.51	1.13	1.41	1.22	1.58	1.21	1.14

	**65**	**66**	**67**	**68**	**69**	**70**	**71**	**72**	**73**	**74**	**75**	**76**	**77**			

*x*	–0.57	–0.47	–1.22	1.23	0.86	–0.31	–0.95	–1.04	–1.54	–1.98	–2.43	–0.40	0.07			
*SD*	1.37	1.54	1.19	1.48	1.70	0.87	1.30	1.12	1.16	1.07	0.97	1.36	1.38			

The soccer events evaluated between −1 and −3, and +1 and +3, comprising a total of 32 items, were selected to be analyzed. In Table 3, it is possible to observe the number of athletes who have chosen each negative (A) or positive (B) dimension of the 32 situations. Two items draw attention, numbers 1 (*Not being in good shape*) and 75 (*Lack of group cohesion*), experienced as having a large amount of negative impact for 111 and 100 players, representing 80 and 72% of the total athletes, respectively. Other items that have a high negative impact on performance for more than 50% of the players were: 9 (*Having problems or conflicts with the coach*), 10 (*Having problems or disputes with the teammates*), 35 (*Losing by a dilated score*), and 59 (*Playing injured*). On the other hand, items 6 (*Establishing high goals*) and 40 (*Playing a derby*) were considered as having a high positive impact on the performance for 87 and 82% of the total athletes. The other items that have a high positive impact were: 19 (*Playing at home*), 20 (*Assuming responsibilities inside the team*), 25 (*Some other team wanting to book you*), 32 (*Doing a speed training*), 34 (*Stretching*), 49 (*Self-pressure to play well*), and 52 (*Knowing in advance that you are going to play*).

**TABLE 3:** Number of athletes who have chosen each negative, neutral, or positive dimension of the 32 situations (in yellow the negatives, and in green the positive situations chosen by 50% or more).

	1	2	3	4	6	9	10	15	16	19	20	25	26	28	32	34
−3	111	44	52	47	–	81	76	55	38	–	–	1	1	55	1	–
−2	21	19	34	27	–	14	12	19	38	2	–	–	1	21	–	1
−1	6	27	31	36	–	20	21	37	32	3	–	1	2	37	–	2
0	–	39	14	27	3	14	25	24	25	11	9	20	62	22	11	11
+1	–	4	3	1	2	4	3	–	1	9	14	20	8	–	15	19
+2	–	5	1	–	13	4	1	2	1	30	39	14	15	3	23	20
+3	–	–	3	–	120	1	–	1	3	80	76	82	49	–	88	85
	35	36	37	38	40	48	49	51	52	59	61	63	68	69	73	75
−3	82	35	40	41	1	–	1	2	7	69	45	67	4	2	38	100
−2	9	20	17	19	–	2	–	3	5	24	25	10	4	9	25	18
−1	31	29	33	42	–	5	4	9	3	33	28	16	5	17	47	17
0	10	46	47	35	5	54	2	38	16	2	39	38	75	41	26	2
+1	4	8	1	1	8	20	17	25	5	7	–	2	4	19	1	1
+2	2	–	–	–	11	23	29	25	10	3	–	3	10	25	–	–
+3	–	–	–	–	113	34	85	36	92	–	1	2	36	25	1	–

**FIGURE 2:**
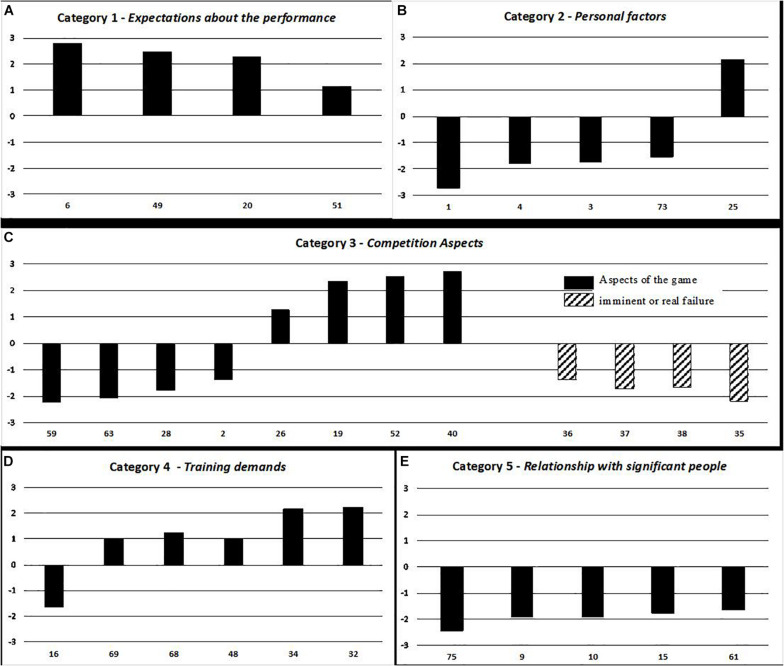
List of soccer event stressors by categories of stress, *Expectations about Performance* **(A)**, *Personal Factor* **(B)**, *Competition Aspects* **(C)**, *Training Demands* **(D)**, and *Relationship with Significant People* **(E)**.

The 32 situations were then classified according to the five categories defined *a priori* (Category 1: Expectations about the Performance, 4 items; Category 2: Personal Factors, 5 items; Category 3: Competition Aspects, 12 items; Category 4: Training Demands, 6 items; Category 5: Relationship with Significant People, 5 items). The 32-item instrument showed good reliability (CR = 0.74). We assessed the CR of each category that presented the following results: CR = 0.65 for Expectations about the performance; CR = 0.51 for Personal factors; CR = 0.64 for Competition aspects; CR = 0.62 for Training demands; and CR = 0.84 for Relationship with significant people. The soccer events are plotted in Figure 2, from the most negative to the most positive impact on the performance.

As shown in Figure 2, a wide variability of the performance’s impact exists among the stress categories. In Category 1, all the events were perceived as positives, and in Category 5, all the events were perceived as negatives. In Categories 2, 3, and 4, some of the events were perceived as negative, while others were perceived as positive. Special attention was given to Category 3 that was divided into three as previously described: opponents, imminent or real failure, and aspects of the game. None of the opponents’ soccer events reached the established result criterion regarding average being from −1 to −3 or +1 to +3.

**FIGURE 3:**
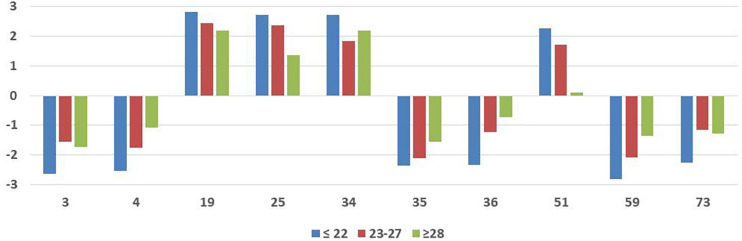
Significant items for younger (≤22 years), average (23–27 years), and older (≥28 years) players.

**FIGURE 4:**
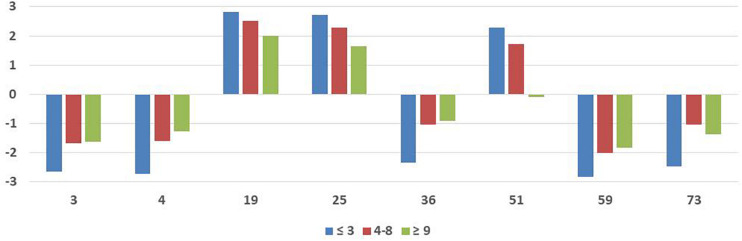
Significant items for less (–3 years), average (4–8 years), and more (+9 years) experienced athletes.

These 32 situations were compared between age (younger, average, and older players), and time as professional (less experience, average, and more experience players). The results by age can be observed in Table 4 and the significant items in Figure 3, and by time as professional in Table 5 and the significant items in Figure 4. Compared by age we can observe differences (*p* < *0.01*) in 10 situations (3, 4, 19, 25, 34, 35, 36, 51, 59, and 73); younger players tend to evaluate situations as more negative or more positive to performance when compared mainly with older players. Compared by time as professionals, we can observe differences (*p* < 0.01) in eight situations (3, 4, 19, 25, 36, 51, 59, and 73) and notice the same trend as before: less experienced players tend to evaluate situations as more negative or more positive to performance when compared mainly with more experienced players.

**TABLE 4:** 32 items for younger (≤22 years), average (23–27 years), and older (≥28 years) players.

		1	2	3	4	6	9	10	15	16	19	20
≥28	*x*	−2.73	−1.00	−1.73	−1.09	2.82	−1.73	−1.73	−1.64	−1.27	2.18	2.27
	*SD*	0.65	1.34	1.01	1.45	0.60	1.35	1.49	1.12	1.35	1.08	1.19
23−27	*x*	−2.80	−1.48	−1.56	−1.76	2.76	−1.88	−1.68	−1.76	−1.44	2.44	2.48
	*SD*	0.5	1.22	1.68	1.22	0.66	1.39	1.31	1.23	1.15	0.91	0.82
≤22	*x*	−3.00	−0.91	−2.64*	−2.55*	3.00	−2.36	−2.45	−2.09	−2.00	2.82*	2.36
	*SD*	0.01	1.76	0.67	0.82	0.01	1.03	0.93	1.04	1.09	0.60	0.92

		**25**	**26**	**28**	**32**	**34**	**35**	**36**	**37**	**38**	**40**	**48**

≥28	*x*	1.36	0.82	−1.18	2.09	2.18	−1.55	−0.73	−1.82	−1.64	3.00	0.82
	*SD*	1.43	1.40	1.53	1.38	1.08	1.29	1.27	0.98	1.12	0.01	1.33
23–27	*x*	2.36	1.36	−1.68	2.12	1.84	−2.12	−1.24¥	−1.48	−1.57	2.76	1.08
	*SD*	1.11	1.70	1.18	1.48	1.46	1.30	1.30	1.36	1.29	0.72	1.32
≤22	*x*	2.73*	1.27	−1.55	2.55	2.73*	−2.36*	−2.33*	−1.89	−1.89	2.45	1.64
	*SD*	0.47	1.35	1.57	0.82	0.47	1.12	1.12	1.17	1.17	1.81	1.43

		**49**	**51**	**52**	**59**	**61**	**63**	**68**	**69**	**73**	**75**	

≥28	*x*	2.82	0.09#	2.64	−1.36	−1,91	−1.64	1.09	1.36	−1.27	−2.27	
	*SD*	0.40	1.81	0.92	1.50	1.04	1.29	1.38	1.75	1.19	1.10	
23–27	*x*	2.40	1.72	2.36	−2.08	−1.48	−2.44	0.64	1.20	−1.16^¥^	−2.56	
	*SD*	1.26	1.24	1.11	1.35	1.56	1.12	1.41	1.29	1.46	0.82	
≤22	*x*	2.91	2.27*	−2.91	−2.82*	−2.09	−2.27	1.64	1.45	−2.27*	−2.73	
	*SD*	0.30	0.79	0.30	0.40	1.22	1.10	1.63	1.44	0.90	0.65	

* difference between ≥28 and ≤22, ¥ difference between 23–27 and ≥28, # difference between ≤22 and 23–27.

**TABLE 5:** 32 items for less (−3 years), average (4–8 years), and more (+9 years) experienced athletes.

		1	2	3	4	6	9	10	15	16	19	20
≥9	*x*	−2.81	−1.09	−1.63	−1.27	2.72	−1.81	−1.90	−1.63	−1.27	2.00	2.27
	*SD*	0.60	1.30	1.02	1.42	0.64	1.07	1.22	1.28	1.34	1.09	1.19
4−8	*x*	−2.76	−1.28	−1.68	−1.60¥	2.76	−2.00	−1.68	−1.76	−1.52	2.52	2.40
	*SD*	0.52	1.24	1.70	1.22	0.66	1.38	1.31	1.16	1.12	0.87	0.86
≤3	*x*	−3.00	−1.18	−2.63*	−2.72*	3.00	−2.36	−2.45	−2.09	−2.00	2.81*	2.36
	*SD*	0.01	1.83	0.67	0.64	0.01	1.02	0.93	1.04	1.09	0.60	0.92

		**25**	**26**	**28**	**32**	**34**	**35**	**36**	**37**	**38**	**40**	**48**

≥9	*x*	1.63	0.81	−1.18	2.09	2.27	−1.72	−0.90	−1.72	−1.63	3.00	0.81
	*SD*	1.43	1.40	1.53	1.37	0.90	1.34	1.44	1.19	1.28	0.01	1.32
4–8	*x*	2.28	1.56	−1.64	2.12	1.80	−2.04	−1.04¥	−1.38	−1.52	2.68	1.08
	*SD*	1.13	1.68	1.41	1.48	1.50	1.30	1.32	1.24	1.20	0.80	1.32
≤3	*x*	2.72*	1.00	−1.81	2.54	2.72	−2.36	−2.33*	−1.88	−2.11	2.45	1.63
	*SD*	0.46	1.26	1.07	0.82	0.46	1.12	1.11	1.26	1.16	1.80	1.43

		**49**	**51**	**52**	**59**	**61**	**63**	**68**	**69**	**73**	**75**	

≥9	*x*	2.72	−0.09#	2.36	−1.81	−1, 8.	−1.72	1.09	1.00	−1.36	−2.36	
	*SD*	0.46	1.57	1.20	1.25	1.16	1.34	1.37	1.54	1.20	0.80	
4–8	*x*	2.40	1.72	2.48	−2.00	−1.52	−2.36	0.84	1.24	−1.04¥	−2.52	
	*SD*	1.25	1.24	1.00	1.38	1.53	1.15	1.37	1.36	1.39	0.96	
≤3	*x*	2.91	2.27*	2.90	−2.82*	−2.36	−2.45	1.18	1.45	−2.45*	−2.73	
	*SD*	0.30	0.79	0.30	0.40	1.03	1.04	1.89	1.44	0.82	0.65	

* difference between ≥9 and ≤3, ¥ difference between 4–8 and ≥9, # difference between ≤3 and 4–8.

A structural model with the 32 items that had the most significant impact (negative or positive) on performance and classified according to the five categories was used to previously evaluate the Field and Indoor Professional Stressors List’s factor structure. Analysis of the players’ responses leads us to believe that the 32 items of the list that had the most impact (negative or positive) on the performance can be explained by two distinct latent variables. The stress items were divided by category; however, within the same category, some items were perceived as eustress while others as distress (although the items were divided by category, it was not possible to conclude based only on the category which one generates stress or eustress). The Composite Reliability value was 0.88 for distress and 0.78 for eustress. Based on the assumption that there is a solution for the set of items with two global factors (distress and eustress) and specific factors (the categories in which the items were classified), a Bi-factorial Exploratory Structural Equation Modeling (BI-ESEM) was employed to assess the factor structure.

The adequacy of the model was assessed by the adjustment meanings Root Mean Square Error of Approximation (RMSEA), Standardized Root Mean Square Residual (SRMR), Comparative Fit Index (CFI), and Tucker-Lewis Index (TLI). According to the literature (Brown, 2015), RMSEA and SRMR values must be less than 0.08, CFI and TLI values must be above 0.90, or preferably 0.95.

The assumption of normality of the data was not satisfied using the Shapiro–Wilk test and the estimation methods were chosen according to the results of the Mardia’s coefficient for data that violate the assumption of multivariate normality (coefficient of multivariate kurtosis = 36.08). The method of extracting the Weighted Least Squares Adjusted by Average and Variance (WLSMV) was implemented in a polychoric data matrix, considering the ordinal nature of the data (Holgado-tello, 2015).

**FIGURE 5:**
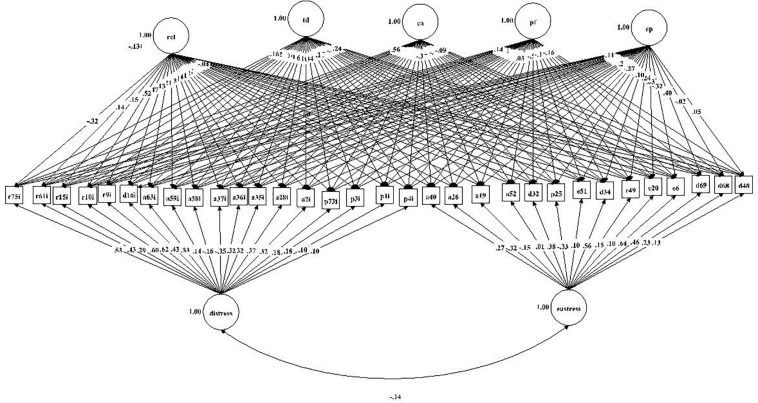
Path diagrams of model. Bifactor solution including global factors (eustress and distress) and specific factors (rel, ac, td, pf, and ep). Note that path diagram figures are only intended to be illustrative as providing detailed labels would make the diagrams too large to present.

The model’s adjustment indexes are acceptable (Chi-Square = 354.77; df = 288; RMSEA = 0.041; CFI = 0.966; TLI = 0.945; SRMR = 0.059). However, the G-factors were not well defined by strong and significant loads (Table 6). For illustration purposes, we present the results of the path diagrams of model in Figure 5.

**TABLE 6:** Standardized factorial loads of the model.

	EP	PF	CA	TD	RE	G-Factor	Uniquenesses
EP06	−0.315					0.642	0.296
EP20	0.037					0.101	0.750
EP49	−0.270					0.157	0.804
EP51	0.511					0.083	0.726
PF01		0.741				0.119	0.209
PF03		0.377				0.150	0.603
PF04		0.271				0.135	0.654
PF25		−0.320				0–367	0.539
PF73		0.389				0.163	0.564
AC02			0.454			0.260	0.682
CA19			−0.025			−0.117	0.576
CA26			−0.120			−0.305	0.579
CA28			0.571			0.323	0.502
CA35			0.665			0.274	0.341
CA36			0.636			−0.359	0.313
CA37			0.705			−0.446	0.126
CA38			0.754			−0.266	0.208
CA40			−0380			0.279	0.676
CA59			0.560			0.140	0.452
CA63			0.628			0.288	0.157
CA52			0.583			0.014	0.014
TD16				−0.090		0.413	0.577
TD32				−0.051		0.365	0.505
TD34				−0.459		0.552	0.421
TD48				−0.266		0.130	0722
TD68				0.542		0.242	0.526
TD69				0.253		0.462	0.450
RE09					0.522	0.622	0.135
RE10					0.568	0.596	0.131
RE15					0.183	0.289	0.649
RE61					0.185	0.432	0.539
RE75					−0.269	0.527	0.218

**EP, expectations about performance; PF, personal factor; CA, competition aspects; TD, training demands; RE, relationship with significant people*.*

In the Relationship with Significant People category, except for item 15, all the others had significant loads above 0.40 in the G-factor. In the Training Demand category, items 16, 32, 34, and 69 were better defined in the G-factor, and item 68 had an acceptable factor load in its respective factor. In the category Aspects of the Competition, items 21, 28, 35, 36, 37, 38, 59, and 63 showed high levels of specificity associated with the S-factor ranging between 0.45 and 0.75 and the other items (21, 19, and 26) presented significant loads, but below the desired level. In the Expectations about the Performance category, items 51 and 6 showed factorial loads of 0.51 and 0.64 in factors S and G, respectively. In the Personal Factors category, except for item 1, all items presented significant loads, however, weak in the respective S-factors, and were poorly defined in the G-factor.

## Discussion

The aims of this study were six, including (a) develop a List of Stressors in Professional Indoor and Field Soccer to facilitate the identification of situations that can cause stress in professional indoor and field players, (b) confirm if the stressful situations can be perceived as both distress and eustress, (c) identify the situations perceived as distress and eustress, (d) check which items make up the five previously established categories, (e) examine the differences in the perception of the situations by age, and time as professional, and (f) assess the factor structure of the list.

The conception considered initially for elaborating the list of stressful situations postulated that the level of stress of a soccer player, indoor or field, is expressed by the intensity of impact on the performance caused by events in the athletes’ sportive lives. Another important point is that the events are not positive or negative *per se*. Still, these soccer events are objective occurrences experienced by the players with enough intensity to impact their performance. During training and competitions, soccer players face a series of requirements, called stressors, which can vary in terms of the content and intensity of their effect on sports performance. Nevertheless, training and competitions are only a part of the player’s sporting experience; according to Noblet and Gifford (2002), other events directly and indirectly related to them, such as family, contract negotiations, transfers, injuries, and rehabilitation, also need to be considered in the investigation of the sources of stress. So, the list was formed by situations that can be classified as (a) inherent in the competitive process, that is, that are directly part of the competition process, are related to the individual or the environment, and (b) adjacent to the competitive process, which happen in everyday life and are independent of the competitive process, such as family problems (De Rose Junior et al., 2004).

Our study confirms that soccer is a challenging environment composed of a spectrum of inherent and adjacent situations, which can impact the performance of the players in a positive (eustress) or negative (distress) way depending on how athletes perceive the situations. In summary, it refers to the extent to which soccer players label the intensity of the cognitive and somatic symptoms of stress experienced in a debilitating-facilitative continuum. This agrees with the studies made by Pargman (1986), Anshel (1990), Jones et al. (1990, 1993), Samulski and Chagas (1992, 1996), Jones and Swain (1995), Jones (1995b), Ntoumanis and Biddle (1998), Brandão et al. (2002), Aschbacher et al. (2013), Kung and Chan (2014), Hargrove et al. (2015), Madigan et al. (2017), McCormick et al. (2018); and have shown that it is not enough to analyze whether certain situations generate stress or not, but mainly, analyze the directional perception of the situations, that is, the nature of interpretation of situations in terms of having a positive or negative relationship with the subsequent performance.

Thus, it is not surprising that the stress phenomenon in sport has been considerably studied in the past decades since high performance sport is characterized by a demand to perform at optimal levels in often intense pressure situations that can interfere with the athletes’ actions, thoughts, and feelings (Brandão, 2000). By its nature, high-performance sport is highly competitive and invariably generates stress in athletes. To Scanlan et al. (1991), with rare exceptions, elite athletes experience stress during their long and arduous sports career to achieve sports excellence, and that would be extremely hard for an athlete to invest so much time and energy in such a challenging environment without feeling any stress or pressure.

However, there is one aspect to consider, as we can observe a large dispersion of responses in some situations (Table 2) which leads us to affirm that the player’s perception in the experience of a particular situation is not uniform, indicating that part of them experience the situation as a performance stimulus and another part as having a negative impact in the performance. These results confirm a classical study of Frester (1976) that considers that equal conditions are often experienced and psychically elaborated differently. This understanding leads to the need for an individual analysis of the subjective perception of the situations.

However, it is interesting to highlight the situations perceived negatively and positively by more than 50% of players (Table 3). The most intensely disturbing situations chosen by players showed firstly how the relationship with significant others can impair the performance of the players, which is in accordance with Ommundsen and Vaglum (1991), Horn (2008), Weinberg and Gould (2008), Kristiansen et al. (2012), and Chan et al. (2019), which studies showed that one of the main sources of negative stress in sport are the reprimands and criticism of the coaches. A poor relationship between the coach and their players, and between players among themselves, can influence the players’ cognitive, emotional, and behavioral processes and how it has consequences in the performance. One player explained that situation: “*Under the command of T., the team lost 5 games in a row. Explosive and even fearless, he soon became incompatible with the team.”*

Secondly is the decrements in performance that arise from playing under condition of injury or not being in a good shape. According to Hägglund et al. (2013), Foster et al. (2001), and Nobari et al. (2020) the workouts’ poorly intensity and volume, the inadequate total load of daily training, training monotony, strain, and accumulated fatigue can lead to injuries and poor physical conditions and interfere on the players’ perceived ability, resulting in feelings of pressure. But a special personal disturbance factor, coming into play injured, despite being considered a debilitating item, appears to be a routine for soccer players. A player’s narrative can show this situation: “*I have suffered a sprain in my right ankle in a dispute with opposing defenders ten days ago, but even so I continued to play.”* Conflictive situations like that can interfere with the player’s physical/psychic balance and game behavior, contribute to burnout and some kind of “hiding” behavior in practice (Semerci, 2019), and consequently, activity engagement is impaired when there are conflicts (Chan et al., 2019).

Thirdly is a fact inherent to soccer concerns to the ongoing or definitive score of a game. Losing from a special score seems like a difficult time that can decide future performance in games. Being imminent or real failures, these stressful situations during competitions provoke psychological disorders and may negatively affect the athlete’s performance (Bailey et al., 2010; Bennett and Maynard, 2017; Brown and Fletcher, 2017). According to Bransen et al. (2019) soccer players are often confronted with in-game situations that could affect their performance, which attest to the need to have a special look at the relationship between certain stressful situations and performance.

However, the most intensely stimulating situations showed that the possibility of success is the motivational key for a soccer athlete. Elite athletes indicate that perceiving success increases their desire to continue practicing the sport, the desire to try harder, and the actual level of effort employed. In this sense, the goal refers to the athlete’s ambition to achieve a certain sporting result. His pressure indicates performance expectations, pressure to meet the established goals, and “positive obligation” to win a certain game and achieve expected results. These agree with Scanlan et al.’s (1989) studies and is confirmed by a narrative of a player: *“I am very critical of myself and I always want to play well. After the game, I analyze everything I did and try to get even better.”* But it is interesting to note that according to Cohn (1990) and Woodman and Hardy (2001) from a cognitive point of view, the perception of pressure from the player to perform well implies great tension on him to the extent where the demands of the sport can exceed the rewards. Our results show the opposite: self-pressure items were evaluated as positive factors, therefore, a performance facilitator.

Specifically, in our study some stressors from the in-game demands have been linked with positive emotions. Traditionally in soccer it is expected that since a team plays in their own “home,” they will already have an advantage in terms of results because they have the support of the fans. Coaches and players believe in the advantage of playing at home (Snyder and Purdy, 1985; Arboix Alió et al., 2020), and about that one player said, *“The pressure of the spectators at home usually influences the performance of the team, which falls in production when is not supported.”* Diana et al. (2017) evaluated the game structure in different contexts (home and away) and showed that the game location strongly influences the game tactics. Another stressor, especially for Brazilian players, is related to positive emotion when playing a derby. Certain games are important by their own characteristics. Because of their impact, significant events have special meanings for players, and playing a derby is almost always associated with memorable memories (Brandão, 2000) as it involves situations with great expectation of performance, experienced as decisive. Beyond that, a “derby” in soccer is a match that mobilizes players, coaches, fans, and the press, even if its result does not interfere with the team’s position on the championship. The following assertion illustrates its importance: “*A derby has a shirt, players, mystique and fans.”*

It is well known in the literature that strength, power, and speed make a difference in the physical (and tactical) performance of soccer players. The determinant abilities in a soccer match involve high demands of speed, agility (change of direction without previous knowledge about the local where the change of direction would be done, involving decision making), and change of direction (in this case, the athletes know the exact time and local of the change of direction), as signaled by Polito et al. (2017). Furthermore, in soccer, speed is a physical problem, but it also involves decision making that will lead to movements. Thus, speed can influence the game’s outcome (Ekblom, 1995) and it is perceived by the players: *“A player who has great speed, has a fundamental characteristic for the game tactical scheme.”*

One aspect that was considered highly positive by the athletes was to know in advance that they are going to be titular. For Lachman (1974) stimulus that is unexpected or suddenly introduced can generate negative emotional reactions. Being warned that they will be a titular minutes before the game require them a quick adaptation and a mental preparation to the new situation according to the demands of unexpected situations: *“The undefinition of the coach about who is going to play often brings a certain emotional instability to the group.”* However, some coaches prefer to decide or disclose the squad at the time of the game, which can generate negative feelings and behaviors, as said one player: “*When the player does not know if he will play, he has 2 thoughts, one positive and one negative. He thinks: if I am going to play, I pay attention to the pep talk before the game. But if he thinks: I can stay out, he doesn’t even care about the game. So, if he finds out in the locker room that he is going to play, it takes him by surprise.”* According to Brandão (2000), ideally, the player should be informed in advance that he is going to play, so that he can prepare himself and have an appropriate mental tactic for the game. Besides, he should know his opponent’s level of performance, physical and mental skills and disabilities, and tactics: “*It is always better to know in advance that you are going to play, you can prepare psychologically better for the game,”* said one of the players.

Apitzsch (1995) stated that the perception of individuals from the external world is not objective but is influenced by subjective interpretations derived from past experiences. According to him, the experience is usually linear with age and with time as professional, meaning that physical, technical, tactical, and psychological skills should increase as a result of increased practice in training and competitions. Younger athletes with little competitive experience do not know the different moments and situations they will face. As a result, they tend to respond to stimuli from the environment differently from more experienced athletes, who have experienced identical or similar situations. Based on this premise, we sought to investigate the differences in the perception of stressful situations according to age and time as a professional, testing the hypothesis that there would be a difference due to these categorical variables. Older and more experienced athletes were expected to perceive situations as less negative to sports performance.

Our results first confirmed the hypothesis and are in accordance with studies conducted by Mahoney et al. (1987) who observed that the most experienced athletes had better concentration before and during competitions, were more self-confident, had a high capacity to recover from errors, experienced less stress before and during competitions, and interpreted anxiety as a facilitator unlike the less experienced (*“This is my first derby against Palmeiras as a professional. I was sleepless in the face of such a responsibility”*), who tended to interpret anxiety as more debilitating to performance (*“Some players lost their emotional balance at the wrong time. Lack tranquility, calm, it’s a very young team”*). Gould and Krane (1992) showed that experienced athletes have different standards when compared to less experienced athletes in terms of stress reactions; Régnier et al. (1993) studied those who have a systematic difference in how increasingly experienced athletes perceive and respond to different environmental stimuli; Brandão (2000), when assessing more experienced soccer players, noted that they evaluated competition situations as less unfavorable to performance than players with less experience. Evidence suggests that, based on previous experiences, more experienced athletes subjectively estimate the probabilities of events and react to them less negatively (“*Insecurity only gets hold of the athlete when he doesn’t have enough experience to believe that he knows how to play”*). The knowledge of the probability of the different events in the sports environment is for Abernethy (1993) a great advantage for the most experienced athletes.

Identifying the antecedents of stress in sport has been an important area of investigation for both theoretical and practical reasons (Ruiz et al., 2019). Familiarity with the categories of stress that are supposed to influence athletes’ emotions and can trigger negative or positive responses will clarify the concept of stress and identify which situations potentially depress performance and which one facilitates performance. Still, it will also help in planning the regime of physical, technical, and tactical training and the preparation of players for the competitive process. Furthermore, when stress categories are chronic negative this can result in “burnout,” lack of motivation, poor sports performance, and even abandonment of competitive sport (Anshel, 1990). A special aspect to be addressed at this point is how the perception of stressful situations also can be different depending on the player’s culture, as analyzed by Brandão et al. (2013). This perception opens an avenue of interesting studies for colleagues from other countries who want to assess the perception of stress in their players and compare it with the Brazilian players.

Related to the List of Stressors in Soccer’s factor-structure, although the model had satisfactory adjustment rates, not all items loaded the respective S-factor or G-factor as strongly as expected. Some of the estimated factor loads for this model proved to be insignificant, which is consistent with the nature of the two-factorial models, in which each item cannot realistically be assumed to have equally strong associations with global and specific factors (Morin et al., 2016). Specific patterns of significant versus non-significant loads may help us to interpret specific items and those that should be classified as stress and/or distress. Future research should look for ways to refine the items designed to improve stress assessment and test them on a large sample to increase this list’s significance.

In conclusion, the results revealed that Brazilian professional soccer players in this study experience various stressful situations. These events are important representations of environmental demands and could predict the performance as they are perceived as distress or eustress. Some of these stressful situations are inherent to the sport environment, and others are adjacent to the sports system or environment. Coach pressure to win and conflicts with teammates are examples of stressors within the team. Family problems and conflicts with press or fans are examples of stressors external to the team, also called peripheral opponents, and showed the relative social influence of significant others in soccer performance.

It is important to highlight that the *List of Stressors in Professional Indoor and Field Brazilian Soccer* was elaborated to fit a specific sport, soccer, indoor and field, and use it to observe the debilitating or facilitating character of stress factors. Knowing the directionality of a given stress factor has important practical implications in preparing athletes and helping them face the performance stressors that are part of the daily life of soccer. This knowledge can also help future athletes and coaches minimize the factors considered negative, which play a critical role in the appearance of psychological and psychosocial disorders and strengthen the positive ones, which significantly impact the players’ motivation.

In this sense, it is believed that the perception of stressful situations and the intensity they affect players behavior should be examined as the players’ set of psychosocial situations; individual experiences in terms of training, team’s social relationships, contract negotiations, player transfers, in addition to competitive and non-competitive aspects and the team’s physical, social, and cultural factors in which the player plays, their infrastructure, media attention, and player support. Furthermore, in a broader sense, in the historical and contemporary contexts in which the athletes are involved, they cannot be satisfied only with this knowledge, but rather convert it into practical training for stress control.

An important delimitation for this study is the following: the small number of futsal players in relation to soccer players, due to the pandemic, so it was not possible to test the hypothesis that there would be no difference in the perception of stress due to this categorical variable.

## Data Availability Statement

The raw data supporting the conclusions of this article will be made available by the authors, without undue reservation.

## Ethics Statement

The studies involving human participants were reviewed and approved by Comitê de ética em pesquisa Universidade São Judas Tadeu. The patients/participants provided their written informed consent to participate in this study.

## Author Contributions

MRFB and LFP conceived the original idea of the study. VH, MC, APM, DO, AO, and LM selected the researches and contributed to data processing and analysis. DA analyzed and presented the data. MRFB, DA, and MVBJ wrote and organized the manuscript. All authors reviewed the manuscript and approved the final version for submission.

## Conflict of Interest

The authors declare that the research was conducted in the absence of any commercial or financial relationships that could be construed as a potential conflict of interest.
